# Caregiver-reported adherence to antiretroviral therapy among HIV infected children in Mekelle, Ethiopia

**DOI:** 10.1186/1471-2431-14-114

**Published:** 2014-04-27

**Authors:** Tadele Eticha, Lwam Berhane

**Affiliations:** 1 Department of Pharmacy, College of Health Sciences, Mekelle University, P.O. Box 1871, Mekelle, Ethiopia

**Keywords:** Adherence, Antiretroviral therapy, Caregiver, Children

## Abstract

**Background:**

Adherence to antiretroviral therapy (ART) in children is complicated may be because of many factors such as child characteristics, caregiver and family characteristics, regimen characteristics, etc. Therefore, it is important to identify factors associated with adherence in HIV infected children in order to reduce the risk of developing treatment failure or drug resistance through interventions. This survey was planned to find out the rate of adherence to ART and its associated factors among the children in Mekelle, Tigray region, Ethiopia.

**Methods:**

A cross-sectional survey was conducted in two hospitals in Mekelle: Ayder Referral Hospital and Mekelle Hospital, during the months of February to March 2013. A structured questionnaire was administered to caregivers to assess patient’s adherence.

**Results:**

Out of a total of 193 patients, 83.4% as reported by caregivers were adherent to ART in the past seven days before the interview. On multivariate logistic regression model, it was found that the children whose caregivers were unmarried (AOR = 15.17, 95% CI: 3.36-68.43) and married (AOR = 3.54, 95% CI: 1.23-10.13) were more likely to adhere to their ART treatment than those whose caregivers were divorced/separated. Similarly, children whose caregivers’ age groups of 25–34 (AOR = 22.27, 95% CI: 4.34-114.29) and 35–44 (AOR = 7.14, 95% CI: 1.65-30.95) were more likely to adhere than their counterparts. The major reasons reported by caregivers for missing medicines include: child being depressed (24.4%), drug side effects (16.3%), too many pills (15.5%) and difficulty in swallowing pills (13.3%).

**Conclusions:**

The prevalence of adherence to ART among children was found to be high and comparable to that of other similar setups. Nevertheless, encouraging the fundamental role of caregivers is so significant to improve adherence among those who missed a dose or more and consequently treatment outcomes of children with HIV.

## Background

An estimated 34 million people were living with HIV as of 2011 globally, including 3.3 million children of less than 15 years. More than 90% of these children live in sub-Saharan Africa. Approximately 2.5 million people, including 330,000 children, were newly infected with HIV. The estimated number of people dying from AIDS-related causes worldwide in 2011 was 1.7 million where 230,000 of them were children [1]. The study conducted among HIV-positive children from 2006–2011 at the Felege Hiwot Referral Hospital, Northwest Ethiopia shows that the mortality rate was 4/100 child years of follow up [2].

Thus, there is a critical need to provide antiretroviral therapy for children who become infected despite the efforts being made to prevent such infections. ART has substantially changed the face of HIV infection where it has been successfully introduced. HIV-infected children now survive to adolescence and adulthood [3]. However, non-adherence to ART may lead to suboptimal drug levels, which may result in therapeutic failure, deterioration of the immune system and/or emergence of drug-resistant HIV strains [4]. In addition to directly affecting personal well-being, poor adherence may compromise programmatic and economic efficiency. Many people receiving first-line regimens found that they fail to respond to treatment at an unnecessarily early stage and would therefore require to switch to more expensive, and often unavailable, second-line regimens [5].

Adherence is therefore a determinant of viral suppression and fundamental to successful ART treatment. There is a direct correlation between risk of virologic failure and proportion of missed doses of antiretroviral drugs [6]. Adherence is a complex health behavior which may be influenced by the dosage regimen prescribed, patient and family factors, and characteristics of health care providers [7]. Adherence behavior in children is found to be more complex in comparison to adults. Limited availability of palatable formulations for the young children is especially problematic, and food requirements for some antiretroviral agents make therapies difficult to administer to infants who require frequent formula feeding. Furthermore, children are dependent on adults for administration of medication; thus, assessment of the capacity for adherence to a complex multidrug regimen requires evaluation of the caregivers and their environments, as well as the ability and willingness of a child to take the drug. A child’s adherence to ART is strongly influenced by caregiver(s) and family function. The caregiver physically gives the medicine to children. Barriers faced by caregivers that can contribute to non-adherence in children include: forgetting doses, changes in routine, being too busy, and child refusal. Concerted effort in clinical care and research are urgently needed to support this vulnerable population [7-9]. Therefore, this study aimed at measuring the prevalence and factors associated with adherence to ART among caregivers of HIV-infected children in Mekelle, Ethiopia.

## Methods

### Study setting

This study was conducted in the Ayder Referral Hospital and Mekelle Hospital in Mekelle, Tigray region, Ethiopia. Mekelle, the capital city of the Tigray Regional State, is located 780 km north of Addis Ababa which is the capital city of Ethiopia. ART program was introduced in 2003 as fee service and the free ART program started in March 2005. According to the Government of Ethiopia, about 26,000 children were eligible for ART in 2010. However, relatively few of the eligible children in need of ART had access to HIV care and treatment services, and up to September 2011, only 15,229 children were on ART [10]. The entire number of HIV infected patients on ART in Ayder Referral Hospital and Mekelle Hospital were 946 and 3637 up to 2013, respectively. Out of these patients, 454 were children and 383 of them belonged to Mekelle Hospital. Health facilities provide access to free ART, as well as to livelihood support, psychosocial support and treatment adherence support.

### Study design and participants

A cross-sectional study was performed in the selected hospitals from February to March 2013. Participants in the study were caregivers of children taking antiretroviral drugs and on follow up in the ART units of the selected hospitals during the study period. Caregivers of children receiving continuous antiretroviral therapy for the last 8 weeks before the study in the selected hospitals and in the age group of 3 months to 14 years were included in the study. But caregivers of children terminally found to be ill were excluded from the study. Caregivers of children were surveyed in research assessments in a community setting, but not in the course of routine care. The sample size was calculated using a single proportion sample size formula. The parameters used to compute the sample size were: proportion of ART adherence among HIV-infected children in Addis Ababa, Ethiopia 86.9% [11], 95% confidence level and a 5% margin of error, which provide a sample size of 175. The total sample size was 193 by adding 10% for the non-response rate. A simple random sampling technique was employed to identify the study units using the ART unique numbers from the registration book in each hospital.

### Adherence measurement

Adherence measurement in the Ayder Referral Hospital and Mekelle Hospital was based on caregivers self-reports in the past three and seven days prior to the interview. Adherence was calculated based on the number of pills reported to have been actually taken divided by the number of prescribed pills over the past seven days. Study participants who reported an intake of ≥95% of the prescribed medication were considered adherent and those with a reported intake of < 95% were classified as non-adherent [12,13].

### Questionnaire design

A structured questionnaire to assess adherence to antiretroviral medications and its associated factors was adopted from other similar setups [11,14]. The questionnaire was originally developed in English; then translated into the local language (Tigrigna) and back into English to check the accuracy by an independent translator. To ensure quality of the data, the questionnaire was pre-tested in 5% of the sample size in similar setups before the actual data collection. The questionnaire consists of three parts which include: socio-demographic characteristics (age, gender, ethnicity, religion, educational status and occupational status of the caregiver, age of the child, gender of the child, family size); health care provider/program related factors to adherence; reasons of missing doses.

### Data analysis

Data entry and analyses were carried out using the Statistical Package for Social Sciences (SPSS) version 20.0. Statistical significance was set at p < 0.05. Bivariate analysis was done using cross tabulations and logistic regression to determine the association between each of the independent variables and adherence. The factors with p values, not greater than 0.25 in bivariate analysis were considered for multivariate analysis to determine the factors associated with adherence and assess for confounding and statistical interaction.

### Ethical considerations

The study was approved for ethical issues by the Health Research Ethics Review Committee of College of Health Sciences, Mekelle University. An official letter of co-operation from College of Health Sciences was given to respective hospitals. Prior to data collection, the aim and objectives of the study were explained to the caregivers, confidentiality was ensured and verbal informed consent was obtained in front of the health professionals at ART Clinics. Verbal informed consent was used in our study based on the results of pretest which shows that forty percent of the study participants (caregivers) were illiterate.

## Results

### Sociodemographic characteristics

A total of 193 caregivers of children was included in the study. The majority (62.2%) of the children were male. The mean age of the children was 7.8 (SD, 3.5) years and 42.5% were older than 8 years of age. Most of the caregivers were female (83.4%). A majority (91.7%) of the study participants were orthodox by religion and 91.2% were Tigre by ethnicity. Just over half of the caregivers were married (53.9%) and unemployed (51.3%) while 32.6% were illiterate. More than half (59.6%) of the caregivers had a monthly income less than 500 Ethiopian Birr (ETB) (Exchange rate 1 USD = 18.8 ETB). One hundred and forty seven (76.2%) of the caregivers were biological parents of the children (Table 1).

**Table 1:** Sociodemographic factors of caregiver and children, and their association with adherence

**Characteristics**	**n(%)**	**Adherence status, n(%)**		**Crude OR (95% CI)**	**p value**
		**Adherent**	**Non adherent**		
Age of child					
<3	11(5.7)	10(90.9)	1(9.1)	2.62(0.31-21.87)	0.375
3-5	40(20.7)	34(85.0)	6(15.0)	1.48(0.54-4.12)	0.449
6-8	60(31.1)	52(86.7)	8(13.3)	1.70(0.68-4.25)	0.256
>8	82(42.5)	65(79.3)	17(20.7)	1	
Gender of child					
Male	120(62.2)	102(85.0)	18(15.0)	1.35(0.62-2.90)	0.450
Female	73(37.8)	59(80.8)	14(19.2)	1	
Gender of caregiver					
Male	32(16.6)	29 (90.6)	3 (9.4)	2.12(0.61-7.45)	0.239
Female	161(83.4)	132 (82)	29 (18)	1	
Age of caregiver					
16-24	18(9.3)	14(77.8)	4(22.2)	1.02(0.25-4.12)	0.977
25-34	69(35.8)	62(89.9)	7(10.1)	2.58(0.82-8.15)	0.105
35-44	75(38.9)	61(81.3)	14(18.7)	1.27(0.46-3.53)	0.646
>44	31(16.1)	24(77.4)	7(22.6)	1	
Ethnic group					
Tigre	176(91.2)	146(83.0)	30(17.0)	1	
Amara	17(8.8)	15(88.2)	2(11.8)	1.54(0.34-7.09)	0.579
Employment status					
Farmer	7(3.6)	6(85.7)	1(14.3)	2.00(0.14-28.42)	0.609
Merchant	22(11.4)	19(86.4)	3(13.6)	2.11(0.28-15.77)	0.466
Student	7(3.6)	6(85.7)	1(14.3)	2.00(0.14-28.42)	0.609
Gov’t employee	29(15.0)	24(82.8)	5(17.2)	1.60(0.25-10.36)	0.622
NGO	21(10.9)	15(71.4)	6(28.6)	1.60(0.13-5.35)	0.848
Unemployed	99(51.3)	85(85.9)	14(14.1)	0.83(0.37-11.04)	0.416
Other	8(4.1)	6(75.0)	2(25.0)	1	
Marital status					
Unmarried	58(30.1)	53(91.4)	5(8.6)	6.70(2.08-21.52)	0.001
Married	104(53.9)	89(85.6)	15(14.4)	3.75(1.51-9.28)	0.004
Divorced/Separated	31(16.1)	19(61.3)	12(38.7)	1	
Educational status					
Illiterate	63(32.6)	52(82.6)	11(17.4)	1.24(0.38-4.05)	0.717
Read and write	14(7.3)	13(92.9)	1(7.1)	3.42(0.36-32.78)	0.286
Elementary	56(29.0)	50(89.3)	6(10.7)	2.19(0.60-8.04)	0.236
High school	36(18.7)	27(75.0)	9(25.0)	0.79(0.23-2.73)	0.709
Collage/university	24(12.4)	19(79.2)	5(20.8)	1	
Religion of caregiver					
Orthodox	177(91.7)	151(85.3)	26(14.7)	4.15(1.22-14.06)	0.022
Catholic	4(2.1)	3(75.0)	1(25.0)	2.14(0.17-27.10)	0.556
Muslim	12(6.2)	7(58.3)	5(41.7)	1	
Monthly income in ETB					
<500	115(59.6)	97(84.4)	18(15.6)	1.03(0.41-2.55)	0.955
500-1000	28(14.5)	22(78.6)	6(21.4)	0.70(0.22-2.27)	0.550
>1000	50(25.9)	42(84.0)	8(16.0)	1	
Family size					
<5	100(51.8)	83(83.0)	17(17.0)	1	
≥5	93(48.2)	78(83.9)	15(16.1)	1.07(0.50-2.28)	0.871
Relation with child					
Biological parent	147(76.2)	119(81.0)	28(19.0)	1	
Non biological parent	46(23.8)	42(91.3)	4(8.7)	2.47(0.82-7.46)	0.109

OR = Odds Ratio, CI = Confidence Interval, Gov’t = Government, Exchange rate: 1 USD = 18.8 Ethiopian Birr (ETB).

### Medication adherence pattern

It was found that from this survey, 172 (89.1%) and 161 (83.4%) of caregivers reported that greater than 95% of total prescribed doses were taken in the past three and seven days, respectively. The majority (63.4%) of the adherent children were male, while 40.4% of them were greater than 8 years of age.

**Figure 1:**
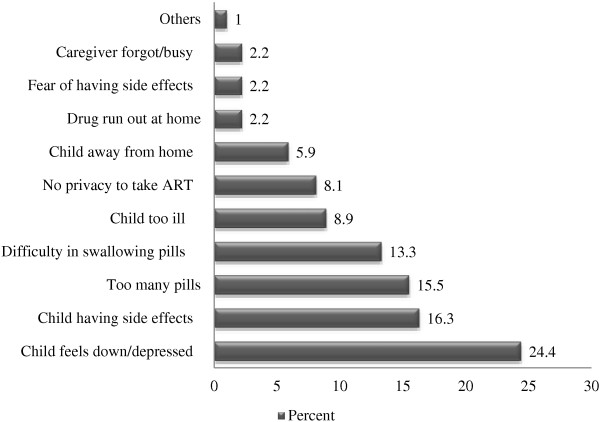
Reasons for missing doses in HIV-infected children.

For those who missed a dose or more, various reasons for non-adherence to antiretroviral medications are displayed in Figure 1. The major reasons cited by the caregivers for missing doses were child being depressed (24.4%), drug side effects (16.3%), too many pills (15.5%) and difficulty in swallowing pills (13.3%).

### Factors associated with children adherence to antiretroviral therapy

Among the sociodemographic factors of caregivers and children, marital status and religion of the caregivers were significantly associated with adherence to ART in children in bivariate analyses. Children whose caregivers were unmarried (OR = 6.70, 95% CI: 2.08-21.52) and married (OR = 3.75, 95% CI: 1.51-9.28) were more likely to adhere to their ART than those whose caregivers were divorced/separated. Similarly, children whose caregivers belonged to the Orthodox religion were 4.15 times (95% CI: 1.22-14.06) more likely to adhere than those whose caregivers belonged to the Muslim religion (Table 1). In bivariate analysis, none of the health provider/program factors were found to be significantly associated with adherence (Table 2).

**Table 2:** Association between health care provider/program factors and adherence

**Characteristics**	**Adherent**	**Non adherent**	**Crude OR (95% CI)**	**p value**
Relation with health care provider				
Excellent	158(83.6)	31(16.4)	1.70(0.17-16.87)	0.651
Good	3(75.0)	1(25)	1	
Frequency of visiting health facility for the child				
Every month	80(80.0)	20(20.0)	1	
Every 2 month	40(88.9)	5(11.1)	2.00(0.70-5.72)	0.196
Every 3 month	28(82.4)	6(17.6)	1.17(0.43-3.20)	0.765
Variable	13(92.9)	1(7.1)	3.25(0.40-26.33)	0.270
Got assistance/information from health care provider				
Yes	161(83.4)	32(16.6)	-	-
No	-	-	-	
Accessibility to reliable pharmacy				
Yes	160(83.8)	31(16.2)	5.16(0.31-84.74)	0.250
No	1(50.0)	1(50.0)	1	
Satisfied with the treatment changes/improvement				
Yes	146(83.8)	29(16.6)	1.01(0.27-3.70)	0.992
No	15(83.3)	3(16.7)	1	
Satisfied with schedule appointment/confidentiality of treatment				
Yes	158(83.6)	31(16.4)	1.70(0.17-16.87)	0.651
No	3(75.0)	1(25.0)	1	

Gender, age, marital status and religion of the caregivers, and caregiver-child relationships were considered for multivariate analysis. After controlling the effects of other variables, age and marital status of caregivers were found to be significantly associated with adherence to ART in children. Children whose caregivers’ age groups of 25–34 (AOR = 22.27, 95% CI: 4.34-114.29) and 35–44 (AOR = 7.14, 95% CI: 1.65-30.95), and children whose caregivers were unmarried (AOR = 15.17, 95% CI: 3.36-68.43) and married (AOR = 3.54, 95% CI: 1.23-10.13) were associated with higher rates of reported adherence as compared to their counterparts. The religion of the caregivers was retained in the multivariate model as cofounders of being unmarried and married caregivers, and caregivers in age groups of 25–34 and 35–44. Age of the caregivers was associated with adherence in multivariate analysis, though it was not significantly associated with the dependent variable in bivariate analyses. Thus, it was found important to control for these variables to validly assess the relationship. The results of multivariate logistic regression analysis are summarized in Table 3.

**Table 3:** Multivariate logistic regression analysis of independent factors and adherence

**Characteristics**	**Adjusted OR (95% CI)**	**p value**
Gender of caregiver		
Male	5.55(0.97-31.68)	0.054
Female	1	
Age of caregiver		
16-24	3.06(0.56-16.84)	0.198
25-34	22.27(4.34-114.29)	0.000
35-44	7.14(1.65-30.95)	0.009
>44	1	
Marital status of caregiver		
Unmarried	15.17(3.36-68.43)	0.000
Married	3.54(1.23-10.13)	0.019
Divorced/Separated	1	
Religion of caregiver		
Orthodox	3.37(0.81-14.03)	0.095
Catholic	1.34(0.06-31.80)	0.857
Muslim	1	
Relation with child		
Biological parent	0.32(0.09-1.19)	0.090
Non biological parent	1	

## Discussion

Adherence to ART in pediatrics is critical in order to maximize the benefit of medication. Inadequate adherence is associated with immunological and virological failure; drug resistance, and treatment failure [15]. In this study, the prevalence of caregivers’ report of ART adherence among children was 89.1% in the past 3 days and 83.4% in the past seven days before the interview. The level of adherence was comparable with those reported in Addis Ababa, Ethiopia, where the prevalence of adherence to ART was 93% in three days and 86.9% in a seven day recall period [11]. Another study conducted at Tikur Anbessa Hospital, Addis Ababa among children on ART reported adherence rate of 93.3% based on caregivers’ report [16]. Similarly, high levels of adherence have been reported from other studies in Tanzania [15], Nigeria [17], Malawi [18], Jamaica [19] and Uganda [20]. A systematic review of pediatric ART adherence revealed that caregiver-reported adherence rates was ranged from 79.5% to 100% in low- and middle-income countries [21]. This suggests that these areas have similar setups in providing ART services to HIV-infected children.

There are different methods to measure pediatric ART adherence, including self- or caregiver-reports, pill counts, pharmacy records, clinic attendance, therapeutic drug monitoring, directly observed therapy, electronic drug monitoring and viral load monitoring. Of these methods, self- or caregiver-reports of adherence are the most frequently used to measure pediatric ART adherence in resource-limited settings. However, these methods overestimate adherence levels and caregiver-reported adherence is generally higher than self-report estimates. These could reflect biases from using a caregiver’s report, such as social desirability bias or recall bias. Both of these biases could result in falsely inflated adherence estimates [21].

The main problems cited by the caregivers which are responsible for a missed dose in this study were child being depressed, drug side effects, too many pills and difficulty in swallowing pills. However, other studies from resource-limited countries have reported that these factors were not the common barriers to medication adherence. The study conducted in Addis Ababa, Ethiopia shows that the most common reasons for missing dose were lack of medication, the child slept and forgetfulness to give the drugs [11] while the survey conducted in the Aminu Kano Teaching Hospital, Nigeria reported that running out of medication and the inability to purchase, travelling difficulty, forgetfulness, and children sleeping as adherence barriers [17]. Similarly, common reasons reported for missed doses in KwaZulu-Natal, South Africa were financial trouble that prevented caregivers from collecting medication on time, vomiting of medication without re-dosing, incorrect dosing by a caregiver, missed clinic appointments and pharmacy collections, confusion between multiple caregivers, and child refusal or self-discontinuation [22]. This may suggest that there is an expanded access to antiretroviral therapy for child patients receiving treatment in the present survey.

Multivariate logistic regression analysis indicated that marital status and ages of the caregivers were independent factors associated with adherence. However, marital status was not associated with adherence in another study [11]. The possible explanations for the greater adherence among children with unmarried and married caregivers in this study might be due to family support in providing care for their children. The religion of the caregivers was found to have an association with adherence in bivariate analyses, but in multivariate analysis it was found to be a confounder. This result was in agreement with another study, where there was no a significant association between religion of the caregivers and adherence [11]. A child’s adherence to ART is strongly influenced by the caregiver and the successful treatment of a child requires the commitment and involvement of a responsible caregiver [3,8]. In this study, biologic caregivers were not associated with better adherence. Nevertheless, a biologic caregiver may experience a stronger emotional connection with the child and be more motivated to promote better adherence compared with a non-biologic caregiver [8].

The interaction between health care providers and the patients is crucial to treatment adherence. Healthcare provider-patient relationship was not a factor significantly associated with adherence in this study. However, the study conducted in South West Ethiopia has shown an association between healthcare provider-patient relationship and adherence [23]. Failure to find an association between healthcare provider-patient relationship and adherence in this survey could be due to the low sample size. Nevertheless, the result shows that there is a good environment for caregivers and/or child patients to tell what the children felt and about their medication course for health care providers in our setting. All study participants got adequate assistance/information from health care providers in this survey like other study [23]. Health care system barriers also affect adherence, especially a regular and timely supply of medication to patients. An unreliable supply of medications can severely reduce patient adherence rates [24]. However, access to reliable pharmacy, treatment changes/improvement and schedule appointment/confidentiality of treatment were found not to be associated with adherence in this study. Failure to find an association in this study may be due to small sample size and the cross-sectional study design used.

Nevertheless, this survey had some limitations. The main limitation was that the methods used for adherence measurement – caregiver reports, which tend to overestimate the prevalence of adherence. Caregivers might be prone to social desirability bias responding inappropriately to the data collectors. The cross-sectional nature may also hinder the ability to exactly identify the predictor of adherence, unlike a longitudinal design. The associations found cannot therefore be assumed to be causal. The sample size was small and may therefore not have been able to detect important associations. In addition, clinical parameters and in depth medication related factors were not explored.

## Conclusions

The study reveals that the prevalence of caregivers’ report of ART adherence among children was found to be 89.1% in the past three days and 83.4% in the past seven days before the date of the interview and it is comparable to that of other similar setups. Marital status (unmarried and married) and age group (25–44) of caregivers of HIV-infected children were found to be significantly associated with adherence. Caregivers reported that child being depressed, drug side effects, too many pills and difficulty in swallowing pills were the main barriers to treatment. Encouraging the fundamental role of caregivers is so important to improve adherence in children with HIV. Further study is recommended to employ more rigorous study designs to detect significant associations.

## Competing interests

The authors declare that they have no competing interests.

## Authors’ contributions

TE and LB participated in the design of the study and interpretation of the results, and drafted the manuscript. LB carried out the survey. TE performed the statistical analysis. Both authors read and approved the final manuscript.

## Pre-publication history

The pre-publication history for this paper can be accessed here:

http://www.biomedcentral.com/1471-2431/14/114/prepub
